# Editorial: Advancing chemotherapy against protozoan parasites: development and repurposing of therapeutic compounds

**DOI:** 10.3389/fphar.2026.1856197

**Published:** 2026-05-01

**Authors:** Rafael M. Mariante, Fernanda F. Evangelista, Victor V. P. Midlej, Juliana Q. Reimão

**Affiliations:** 1 Laboratory of Structural Biology, Instituto Oswaldo Cruz, Fiocruz, Rio de Janeiro, Brazil; 2 Universidade Federal da Integração Latino-Americana, Foz do Iguaçu, Brazil; 3 Laboratory of Preclinical Assays and Research of Alternative Sources of Innovative Therapy for Toxoplasmosis and Other Sicknesses (PARASITTOS), Faculdade de Medicina de Jundiaí, Jundiaí, Brazil

**Keywords:** antiparasitic compounds, Chagas disease, drug repurposing, *Leishmania*, leishmaniasis, *Toxoplasma gondii*, toxoplasmosis, *Trypanosoma cruzi*

## 1 Introduction

Protozoan parasites are responsible for some of the most debilitating neglected tropical diseases worldwide, including Chagas disease, leishmaniasis, and toxoplasmosis. Together, these infections affect hundreds of millions of people, predominantly in low- and middle-income countries, and impose a substantial burden on public health systems. Despite their medical significance, the therapeutic arsenal available against these parasites remains limited. Current first-line treatments–such as benznidazole and nifurtimox for Chagas disease, meglumine antimoniate and amphotericin B for leishmaniasis, and sulfadiazine/pyrimethamine for toxoplasmosis–are hampered by toxicity concerns, limited efficacy, and the emergence of drug resistance (Capela et al., 2019; Hu et al., 2025). These shortcomings underscore the urgent need for the development of novel and effective therapeutic strategies.

Drug repurposing–the identification of new therapeutic indications for existing compounds–has emerged as a compelling and cost-effective approach to accelerate antiparasitic drug discovery. By leveraging the known safety profiles and pharmacokinetic data of approved drugs, this strategy can significantly reduce the time and resources required for clinical translation (Vijayasurya et al., 2024; Hu et al., 2025). This editorial introduces a Research Topic of four studies that collectively advance our understanding of drug repurposing strategies targeting three major protozoan pathogens: *Trypanosoma cruzi*, *Leishmania* spp., and *Toxoplasma gondii*.

## 2 Amiodarone in chagas disease: from antiarrhythmic to trypanocidal agent

Chagas disease (CD), caused by *T*. *cruzi*, remains a major cause of chronic cardiac disease, with current antiparasitic therapies showing limited efficacy and frequent adverse effects that hinder adherence (Sousa et al., 2024). In this context, Barbosa et al. provide a comprehensive and timely evaluation of the dual role of amiodarone (AMIO) in CD management, integrating bibliometric mapping with a systematic review approach to offer insights into global research trends while critically assessing existing evidence on AMIO’s antiarrhythmic and potential trypanocidal effects. The findings reinforce that AMIO remains widely used to control ventricular arrhythmias in Chagas cardiomyopathy, with consistent reductions in arrhythmic events reported across studies. Importantly, the review highlights emerging evidence suggesting additional antiparasitic and immunomodulatory properties, positioning AMIO as a promising candidate for drug repurposing. However, the authors underscore a major limitation in the field: the scarcity of robust clinical trials and the overall low-to-moderate quality of available evidence, particularly regarding hard clinical outcomes such as mortality and hospitalization. This work exemplifies the challenges and opportunities in repurposing established drugs for neglected diseases, emphasizing the urgent need for well-designed clinical studies and combination therapies to fully elucidate the therapeutic potential of AMIO in CD and determine whether its antiparasitic and anti-inflammatory effects translate into meaningful clinical benefit.

## 3 Memantine as a repurposed oral therapy for visceral leishmaniasis

In a field where available therapies for visceral leishmaniasis remain constrained by toxicity, high cost, and limited oral bioavailability (Zhang et al., 2025), Gonçalves-Ozório et al. investigate memantine, an N-methyl-D-aspartate (NMDA) receptor antagonist approved for Alzheimer’s disease, as a repositioned candidate for visceral leishmaniasis–a potentially fatal neglected disease. The study is notable for demonstrating dual activity: direct leishmanicidal effects against intracellular amastigotes *in vitro*, with a high selectivity index, and significant reduction of hepatic and splenic parasite burden in a murine model at oral doses that outperformed meglumine antimoniate under certain conditions. Mechanistically, parasite clearance was associated with a shift toward Th1-dominant immunity and activation of Th2 and Th17 pathways, suggesting host-directed immunomodulation independent of nitrosative stress as a key component of its mechanism of action. These findings are particularly relevant given the oral route of administration and the favorable safety profile observed across experimental conditions. The authors recognize, however, that the study is limited to preclinical murine models, and that the translational gap to human disease remains to be addressed through clinical investigation. This work reinforces drug repositioning as a viable strategy for accelerating the development of safe and accessible oral therapies for visceral leishmaniasis.

## 4 18-Methoxycoronaridine: an oral alkaloid with translational antileishmanial potential

The study by Delorenzi et al. investigates 18-methoxycoronaridine (18-MC), an iboga-type indole alkaloid originally developed as an anti-addiction agent, as an oral therapeutic candidate against *Leishmania amazonensis*–a species responsible for American tegumentary leishmaniasis, for which effective treatments remain a critical unmet need (de Vries and Schallig, 2022). Using both murine and non-human primate models, the authors provide an unusually comprehensive translational evaluation integrating efficacy, pharmacokinetics, and toxicological profiling across species. Short-course oral treatment achieved greater than 99% parasite burden reduction in mice, while non-human primates demonstrated a clear dose-dependent response with up to approximately 98% lesion reduction and marked tissue re-epithelialization. Importantly, pharmacokinetic-pharmacodynamic modeling revealed a strong exposure-response relationship and a favorable therapeutic index, supporting translational relevance. Safety assessments indicated acceptable tolerability across species, with a defined no-observed-adverse-effect level (NOAEL). This multi-species approach is a methodological strength that distinguishes this study from most preclinical antileishmanial work, which rarely extends beyond rodent models. Collectively, this work advances 18-MC as a compelling oral candidate and highlights the translational value of integrating pharmacokinetic modeling and cross-species efficacy assessment in antiparasitic drug development.

## 5 Novel MMV pathogen box compounds against *T*. *gondii* at the maternal–fetal interface

de Oliveira et al. present the first evaluation of Medicines for Malaria Venture (MMV) Pathogen Box compounds in experimental models of the maternal-fetal interface, directly addressing a critical gap in the search for safer therapies against congenital toxoplasmosis–a condition for which current treatment with sulfadiazine and pyrimethamine carries well-documented risks of bone marrow suppression and teratogenicity (Dunay et al., 2018). The three tested compounds–MMV675968, MMV022478, and MMV021013 – demonstrated antiparasitic activity at non-cytotoxic concentrations in both trophoblastic cells and placental explants, with irreversible inhibition of parasite proliferation confirmed in the cellular model and activity profiles broader than pyrimethamine, including interference with early lytic cycle stages such as adhesion and invasion. MMV021013 emerged as the most promising candidate, distinguished by predicted blood-brain barrier permeability and a clean toxicity profile–a particularly relevant feature given that *T. gondii* establishes persistent cysts in cerebral tissue and that both placental and CNS barriers share key physicochemical properties. The authors acknowledge that the exclusive use of *in vitro* and *ex vivo* models and short treatment periods preclude conclusions on long-term efficacy or resistance development, calling for *in vivo* validation as the critical next step toward clinical translation of these candidates.

## 6 Concluding remarks

The studies assembled in this Research Topic collectively illustrate the breadth and promise of contemporary strategies to advance antiparasitic chemotherapy against protozoan pathogens, including *T*. *cruzi*, *Leishmania* spp., and *T*. *gondii*. From the reassessment of an established cardiac drug (amiodarone) to the preclinical validation of a repurposed Alzheimer’s therapy (memantine), the translational characterization of a plant-derived alkaloid (18-MC), and the identification of MMV Pathogen Box candidates targeting *T. gondii* at the maternal-fetal interface, these contributions represent meaningful steps toward expanding the therapeutic pipeline for neglected tropical diseases and other globally relevant parasitic infections. Together, they underscore the value of drug repurposing, and rigorous translational design in overcoming the limitations of current treatments. We hope this Research Topic will stimulate further mechanistic studies, encourage clinical translation, and foster collaborative efforts to address the unmet needs of the millions affected worldwide.

## References

[B1] Capela R. Moreira R. Lopes F. (2019). An overview of drug resistance in protozoal diseases. Int. J. Mol. Sci. 20 (22), 5748. 10.3390/ijms20225748 31731801 PMC6888673

[B2] de Vries H. J. C. Schallig H. D. (2022). Cutaneous leishmaniasis: a 2022 updated narrative review into diagnosis and management developments. Am. J. Clin. Dermatol 23 (6), 823–840. 10.1007/s40257-022-00726-8 36103050 PMC9472198

[B3] Dunay I. R. Gajurel K. Dhakal R. Liesenfeld O. Montoya J. G. (2018). Treatment of toxoplasmosis: historical perspective, animal models, and current clinical practice. Clin. Microbiol. Rev. 31 (4), e00057-17. 10.1128/CMR.00057-17 30209035 PMC6148195

[B4] Hu S. Batool Z. Zheng X. Yang Y. Ullah A. Shen B. (2025). Exploration of innovative drug repurposing strategies for combating human protozoan diseases: advances, challenges, and opportunities. J. Pharm. Anal. 15 (1), 101084. 10.1016/j.jpha.2024.101084 39896318 PMC11786068

[B5] Sousa A. S. Vermeij D. Ramos A. N. Jr Luquetti A. O. (2024). Chagas disease. Lancet 403 (10422), 203–218. 10.1016/S0140-6736(23)01787-7 38071985

[B6] Vijayasurya G. S. Shah S. Pappachan A. (2024). Drug repurposing for parasitic protozoan diseases. Prog. Mol. Biol. Transl. Sci. 207, 23–58. 10.1016/bs.pmbts.2024.05.001 38942539

[B7] Zhang H. Yan R. Liu Y. Yu M. He Z. Xiao J. (2025). Progress in antileishmanial drugs: mechanisms, challenges, and prospects. PLoS Negl. Trop. Dis. 19 (1), e0012735. 10.1371/journal.pntd.0012735 39752369 PMC11698350

